# Supplementary material for: Precise tea leaf disease detection using UAV low-altitude remote sensing and optimized YOLO11 model
Source: PLoS One. 2026 Feb 18;21(2):e0342545. doi: 10.1371/journal.pone.0342545 (PMC12915959; doi:10.1371/journal.pone.0342545)
Supplement: Attachment — Submitted filename: PONE-D-25-19024_Review#OB.pdf [file pone.0342545.s001.pdf]

Manuscript title: Precise Tea Leaf Disease Detection Using UAV Low-Altitude Remote Sensing and Optimized YOLO11 Model

Manuscript ID: PONE-D-25-19024

Journal: PLOS ONE

Article type: Research paper

Content language: English

Keywords: FCHE-YOLO model, YOLO11 model, UAV, Tea leaf disease detection, Lightweight deep learning

The manuscript addresses an important and current challenge in agricultural monitoring achieving accurate and real-time detection of tea leaf diseases through the integration of low-altitude remote sensing via Unmanned Aerial Vehicles (UAVs) and an enhanced, lightweight deep learning framework, namely the FCHE-YOLO model, which is an optimization of the YOLO11 architecture. The study is technically well-founded and represents a meaningful contribution to the ongoing development of UAV-assisted plant health monitoring systems, which are critical for promoting sustainable agricultural practices and mitigating crop losses due to pests and diseases.

The study is technically competent and makes an important contribution to the continuous development of UAV-assisted plant health monitoring systems, which are vital for encouraging sustainable agricultural practices and decreasing crop losses due to pests and diseases. Overall, the paper is clearly written, logically structured, and supported by a robust set of experimental results that demonstrate the potential benefits of the proposed improvements. Nevertheless, in order to meet the publication standards of *PLOS ONE* and to maximize the impact and reproducibility of the work, several aspects require further elaboration.

Major comments

- The manuscript introduces a novel backbone module (FC_C3K2), a redesigned neck structure (HSFPN), and a lightweight detection head (Efficient-Head), all integrated into the YOLO11 framework. While these modular enhancements are technically reasonable and well-supported by the experiments, the degree of incremental novelty compared to recent lightweight YOLO versions, such as YOLOv8 or other contemporary architectures, is not yet clearly demonstrated.
  To strengthen the contribution and improve clarity for readers, I suggest the authors to provide a more rigorous discussion quantifying and explaining how the proposed FCHE-YOLO architecture differs both functionally and conceptually from existing lightweight variants, including examples like YOLOv8-RCAA and YOLO-Tea. Additionally, presenting a comparative table that outlines the module-wise differences would greatly help to highlight the specific innovations introduced in this study.
- The dataset used in this study consists of approximately 5,400 images obtained through data augmentation, all originally collected from a single tea plantation (Siwangshan). This limited source of data raises valid concerns regarding the potential for overfitting and questions the generalizability of the model to other locations and conditions. Moreover, The data augmentation techniques could be described with precise parameter ranges (e.g., how much brightness adjustment? what rotation angles?).
  I suggest the authors to provide a thorough discussion of possible site-specific biases and clarified whether data from additional plantations, different seasons, or varying lighting conditions were included in the dataset or are planned for future work.
- The comparative experiments presented in the manuscript primarily concentrate on conventional models such as Faster R-CNN, SSD, and earlier versions of the YOLO series. However, the recent advancements in transformer-based or hybrid CNN-transformer detection frameworks, such as DE-TR and Swin-Transformer, are not addressed or evaluated in this study.
  I recommend the authors to discuss why transformer-based or attention-heavy detectors were not compared or tested. Even if not deployed on UAVs due to resource constraints, this would contextualize the chosen architecture.
- Although the model demonstrates high frame rates on a desktop GPU, its performance decreases to approximately 15 FPS when deployed on edge UAV devices, which could limit its real-time effectiveness in the field. While the manuscript briefly mentions plans for model pruning and TensorRT optimization, I recommend authors to include more specific details on current inference latency and energy consumption during UAV operations. Providing this information would offer a clearer understanding of the model's practical feasibility and highlight any technical constraints that need to be addressed for successful real-world deployment.
- Many hyperparameters are listed, but key training details such as random seed, augmentation pipeline code, and training/validation split reproducibility are missing. I recommend authors to consider adding the full training pipeline and data splits to a supplementary file or public repository to meet PLOS ONE's data availability standards.

Minor Comments

- The manuscript has minor grammatical issues and redundant phrases. For example, “the model significantly improves the adaptability and expressiveness” could be shortened, in addition, some references are embedded mid-sentence and break the flow
  I Recommend authors a careful language editing and to ensure consistent citation style;
- Figures illustrating the system architecture and modules should be labeled more clearly and be of higher resolution. Some figures (heatmaps, detection examples) are low-resolution and need clearer labeling.

I recommend to use high-resolution annotated figures with consistent color maps and scale bars.

- Some cited papers (e.g., [20], YOLO-Tea) should be cross-checked for accuracy and updated if more recent works exist.

Final Recommendation

The study presents an innovative approach for precise tea leaf disease detection by integrating UAV low-altitude remote sensing with a lightweight, optimized YOLO11-based model. It demonstrates clear advances in balancing detection accuracy, model compactness, and practical deployment on edge devices, which is highly relevant for precision agriculture and field-based plant health monitoring.
However, minor revisions are necessary to strengthen the manuscript’s scientific rigor. Key areas for improvement include providing a clearer discussion of the model’s generalization limitations, adding class-wise performance metrics and confusion analysis, clarifying inference speed bottlenecks on UAV hardware, and positioning the proposed feature fusion strategy relative to existing architectures like PANet or BiFPN. Additionally, minor language and structural edits will improve clarity and readability.

With these revisions, the manuscript will make a solid contribution to UAV-based crop monitoring and lightweight deep learning applications in agriculture. Therefore, I recommend **Minor Revision** before acceptance for publication.
