# Supplementary material for: Precise tea leaf disease detection using UAV low-altitude remote sensing and optimized YOLO11 model
Source: PLoS One. 2026 Feb 18;21(2):e0342545. doi: 10.1371/journal.pone.0342545 (PMC12915959; doi:10.1371/journal.pone.0342545)
Supplement: Attachment — Submitted filename: Review_PONE-D-25-19024.pdf [file pone.0342545.s002.pdf]

The abstract of the paper needs a revision. It needs to be precise and must convey the summary and not the details of each method.

The images are of low quality. They need to have clear labels, with words spelled out fully instead of just abbreviations.

The figures throughout the paper convey too less information. They need to be self-explanatory and elaborative.

You must provide full names before abbreviations throughout the paper.

The paper's English can be improved. It is suggested to get it proof read.

The organization of the paper can be improved and information shared systematically.

Introduction

- You have mentioned that the current methods require high performance hardware, and follow up with the proposal of deep learning methods which has even higher processing requirements.

Materials

There distinction and information the authors have include din the materials and Methods sections are actually interlinked. Combine the two titles to make it Materials and Methods instead of having separate sections.

- The image quality of Figure 1 is poor. I was unable to read it even after downloading it. Also, the abbreviations need to be defined for a reader to understand the process. The backend system also needs to be elaborated upon. This need to be observed for all the images.
- In the hardware system, one of the core items are the cameras. Provide further explanation and use of multiple cameras.
- What are your criteria, justification and reasoning of selecting this particular field and planning the path as defined? What are you referring to when you say UAV flew at 10-meter intervals? It is not clear.
- It is not clear to me if the drone was custom built or off the shelf?
- What was the purpose and use of other sensors in your study as you have defined in your DJI selected drone, Table 1.
- The drone seems to be significantly large with 26kg weight. How this aligns with your claim of compact size drone. These two seem to be contradictory.
- What was the height of your drone flight? It has not even been represented in your Figure 4.
- How frequently images were captured and what was the reasoning behind this interval?
- I would suggest to create a table and define the key parameters of your flight and planning.
- In the start of your paper, you have stated mainly two types of tea plant diseases, but then in your image selection section, you have stated more than 8 type of diseases. This makes the study a lot more complex. You must state clearly what is the scope of your study in the start and stick to the plan instead of taking into account additional factors for your study to be focused.
- Why 3,000 images were captured, why this number.

Methods

- Explain the role of every method you have used and align it with your research questions and also the function you are achieving in a summary table to show how these different methods come together to achieve an overall outcome.
- The network structure you have presented in Figure 6 for YOLO is already quite complicated for a general reader. You must provide a high-level overview with clear names and labels before diving into a detailed structure.
- You are using abbreviations in heading which should usually not be the case. Abbreviations must be used in the text body and not the headings unless they are already defined.
- Within your proposed structure in figure 7, you must explain the logic behind the presence of repeated FasterNet Blocks.

Experiments and Analysis

- Authors mentioned section 3.2 but where is it in the paper?
- Results in Table 4 must highlight the top three models. Label your model as (Our) in the table along with the name.
- Provide F1 score results for the combined precision and Recall.
- What was the basis of selecting various models for comparison. Full justification nee dto be provided.
- Radar Figure 18 must be further elaborated upon.
- You have not provided any experimental conditions for UAV testing, and type of tests conducted + how many tests.
- Why only heat maps of two models were selected. Heatmap of all the comparative models in your study should be compared and discussed.

Further experimentation results are required as stated above.
