# Supplementary material for: Precise tea leaf disease detection using UAV low-altitude remote sensing and optimized YOLO11 model
Source: PLoS One. 2026 Feb 18;21(2):e0342545. doi: 10.1371/journal.pone.0342545 (PMC12915959; doi:10.1371/journal.pone.0342545)
Supplement: Attachment — Submitted filename: Review_PONE-D-25-19024R1.pdf [file pone.0342545.s004.pdf]

Overall, there has been an improvement in the paper but some further enhancements and modifications are required, or at least responses should be separately provided to some of the questions that are not addressed within the paper with justifications. Also indicate where you have carried out changes by taking each comment and responding to it in a document.

The paper's English can be improved. This has not been fully addressed as there are some grammatical error.

Materials and Methods

- In the start of the section, only a brief intro of the backend system will be sufficient. I would suggest to include the detailed discussion on the backend system within the appropriate section, such as by introducing the **software system components section**. This relates to reorganisation of the paper.
- There is an error in the spelling of the caption for figure 4.
- You say that the system was self-developed. Was the whole drone system + detection system was developed by yourself the development took place only at the level of the detection system which was then integrated within the off the shelf drone. This question was raised in my previous review as well. Please state clearly.
- There is a mention of images collection but the phrase suggests images will be collected in the future. If that's the case why the authors have decided to choose 3037 images. If they have already been collected please correct the grammar and also provide the reason for collecting 3037 images. **This question was raised in the previous review.**
- The headings hierarchy is a bit confusing and it is hard to differentiate between the main heading and the sub-heading. E.g. 'Methods' versus 'Yolo11 Network Structure'. Please check and distinguish between them carefully.
- I did not seem to find an answer to this question I raised - **The drone seems to be significantly large with 26kg weight. How this aligns with your claim of compact size drone. These two seem to be contradictory.**
- This has not been addressed from my previous review: **Explain the role of every method you have used and align it with your research questions and also the function you are achieving in a summary table to show how these different methods come together to achieve an overall outcome.** To make it clear, I would suggest authors to include an outline/flow diagram or a table including all the approaches, showing a stage + approach in the flow and the function/purpose against each one of them to provide a bigger picture to the readers.
- The following also relates to my previous comment. **The network structure you have presented in Figure 6 (now 7) for YOLO is already quite complicated for a general reader. You must provide a high-level overview with clear names and labels before diving into a detailed structure.** If you have provided this already, indicate where it is in the paper?

Experiments and Analysis

- Authors mentioned sections 2.2 and 3.2 but where are they in the paper? None of the sections in this paper are numbered. How a reader will know what part of the paper is 2.2 or 3.2, etc.?
- This comment has not been addressed: **Results in Table 4 and other tables must highlight the top three models. Label your model as (Our) in the table along with the name.**

- Figure 17, the x/y axis have not been properly labelled and does not provide explanation of what this figure is about, when looking at the figure itself without referring to the text.
- Table 5, add **(our)** against your model(s).
- The authors may consider finding a better way to represent the images from all the model results (Fig. 22-32) since they are the figures presented one after the other in a row which is a bit odd for the paper structure.
- Indicate where this is in the paper? **Provide F1 score results for the combined precision and Recall.**
