# Supplementary material for: Precise tea leaf disease detection using UAV low-altitude remote sensing and optimized YOLO11 model
Source: PLoS One. 2026 Feb 18;21(2):e0342545. doi: 10.1371/journal.pone.0342545 (PMC12915959; doi:10.1371/journal.pone.0342545)
Supplement: Attachment — Submitted filename: Response to Reviewers.docx [file pone.0342545.s003.docx]

**Response to Reviewers**

The authors would like to express their gratitude to the reviewers for their comments and suggestions on our manuscript entitled "Precise Detection of Tea Leaf Diseases Using UAV Low-Altitude Remote Sensing Combined with an Optimized YOLO11 Model" [PONE-D-25-19024]. These comments are extremely valuable, as they have been of great help in revising and improving the paper and have provided important guidance for our research. After carefully reading the reviewers' comments, we have made substantial revisions to the manuscript and hope that it will be approved.

In the revised manuscript, the responses to the reviewers' comments are marked in red. The following is a detailed explanation of how we have addressed these comments and suggestions in the revised paper.

Reviewer #1

**Major comments**

•	The manuscript introduces a novel backbone module (FC_C3K2), a redesigned neck structure (HSFPN), and a lightweight detection head (Efficient-Head), all integrated into the YOLO11 framework. While these	modular enhancements are technically reasonable and well-supported by the experiments, the degree of incremental novelty compared to recent lightweight YOLO versions, such as YOLOv8 or other contemporary architectures, is not yet clearly demonstrated.

To strengthen the contribution and improve clarity for readers, I suggest the authors to provide a more rigorous discussion quantifying and explaining how the proposed FCHE-YOLO architecture differs both functionally and conceptually from existing lightweight variants, including examples like YOLOv8-RCAA and YOLO-Tea. Additionally, presenting a comparative table that outlines the module-wise differences would greatly help to highlight the specific innovations introduced in this study.

**Reply:** Thank you for your valuable suggestions! We have added detailed comparative experiments with YOLOv8-RCAA, YOLO-Tea, and lightweight models in the paper (see Section 4.4.2), which highlight the advantages of FCHE-YOLO in terms of accuracy and inference efficiency. In addition, to more clearly demonstrate the innovative points of each module, we have added Table 6 in the paper, which systematically compares the differences between FCHE-YOLO, YOLOv8-RCAA, and YOLO-Tea in terms of structural design and deployment feasibility. Through this approach, we further clarify the uniqueness and innovativeness of FCHE-YOLO compared to existing lightweight models at both functional and conceptual levels.

**The detailed details are as follows:**

**Comparison of Algorithm Performance**

To verify the detection performance of the proposed FCHE-YOLO model, several mainstream object detection models are selected for comparative experiments, which cover different types of network architectures, including YOLO series (v5n, v8n, v10n, v11n), Faster R-CNN, SSD, Transformer structure RT-DETR series models, as well as recently proposed improved versions such as YOLO Tea and YOLOv8-RCAA.The reasons for choosing these models are multifaceted.

Firstly, these models have a wide range of applications in the field of computer vision and perform excellently in their respective domains. YOLO series and Faster R-CNN models are classic models in the object detection field, and their excellent performance is proven in multiple practical tasks. Therefore, choosing these models as comparison objects is to fully verify the advantages of the FCHE-YOLO model. Secondly, the comparison with these models occupying different architecture and characteristics benefits to demonstrate the advantages of FCHE-YOLO from multiple dimensions. The YOLO series models are mainly single-stage detection networks, emphasizing real-time performance and inference speed, especially suitable for edge device applications requiring efficient processing; Faster R-CNN is a two-stage detection network that performs well in accuracy, but has slow inference speed and high computational cost; the RT-DETR model adopts the Transformer architecture and exhibits certain advantages in handling complex target relationships, but large computational load makes it unsuitable for running on resource constrained devices. YOLO-Tea and YOLOv8-RCAA as improved models for tea disease detection are incorporated, providing a comparative perspective to validate the advantages of FCHE-YOLO in this specific task.

The above models covering different types of architectures can verify the performance advantages of FCHE-YOLO by comparison from detection accuracy, model complexity, and deployment efficiency. Table 5 presents the comparative results of different object detection models.

**Table 5. Performance Comparison of Different Detection Models**

| Model | P/% | R/% | F1/% | mAP0.5/% | FPS(f/s) | FLOPs/G | Size(MB) |
| --- | --- | --- | --- | --- | --- | --- | --- |
| Faster RCNN | 79.5 | 78.3 | 73.5 | 78.6 | 6.5 | 209 | 149 |
| SSD | 68.1 | 67.3 | 64.7 | 67.7 | 28 | 29.5 | 90.1 |
| YOLOv5n | 89.5 | 86.1 | 85.1 | 88.4 | 35.6 | 6.3 | 14.7 |
| YOLOv8n | 91.5 | 90.6 | 88.9 | 91.2 | 41.3 | 8.9 | 6.23 |
| YOLOv10 | 89.5 | 90.0 | 86.4 | 89.7 | 39.5 | 6.7 | 5.6 |
| RT-Detr | 93.7 | 93.1 | 90.6 | 93.4 | 37.1 | 21.2 | 63.4 |
| YOLO11 | 95.3 | 93.4 | 92.2 | 94.1 | 43.3 | 6.4 | 5.24 |
| YOLOv8-RCAA | 98.2 | 85.3 | 91.3 | 97.9 | 45.6 | 8.1 | 6.46 |
| YOLO-Tea | - | - | **90.4** | 92.3 | 39.4 | 7.9 | 15.6 |
| FCHE-YOLO | **98.9** | **97.8** | **96.0** | **98.1** | **47.5** | **4.2** | **3.04** |

The experimental results show that the improved FCHE-YOLO model performs optimally overall, with mAP 0.5 reaching 98.1%. Compared to Faster R-CNN, SSD, YOLOv5n, YOLOv8n, YOLOv10, RT-Detr, YOLO11, YOLOv8-RCAA and YOLO-Tea models, it shows the mAP is improved by 19.5%, 30.5%, 9.7%, 6.9%, 8.4%, 4.7%, 4%, 0.2% and 5.8% respectively, and the F1 score is increased by 22.5%, 31.3%, 10.9%, 7.1%, 9.6%, 5.4%, 3.8%, 4.7%, and 5.6% respectively.

Specifically, Faster R-CNN has a floating-point operation count of 209 G with a large model size and slow detection speed, failing to meet real-time detection requirements. The single-stage detection model SSD, with a weight of 90.1 MB and detection speed of 29.5 frames per second, struggles to meet the real-time and resource-constrained requirements of UAV devices. Although the RT-Detr model exhibits excellent accuracy, its excessive parameter count makes it unsuitable for real-time detection on edge devices. Compared to the YOLO series algorithms, the improved FCHE-YOLO model performs best in model size, detection speed, and computational complexity, making its overall performance superior. The improved FCHE-YOLO model can meet the requirements for precise detection of tea leaf diseases by UAVs.

471

474

475

476

477

In the YOLO series algorithms, YOLOv5n performs well in speed (35.6 FPS), but its accuracy is relatively low, with a mAP of only 88.4%, inferior to FCHE-YOLO. YOLOv8n has a higher accuracy (mAP of 91.2%), a smaller model size (6.23 MB), and a good inference speed (41.3 FPS), yet there is a gap in accuracy and computational complexity compared to FCHE-YOLO. YOLOv10 achieves a good balance between accuracy (mAP of 89.7%) and speed (39.5 FPS), but still falls short of FCHE-YOLO's performance. YOLO11’s accuracy and inference speed improve to 94.1% and 43.3 FPS, respectively, close to FCHE-YOLO, however its computational complexity (6.4 G FLOPs) and model size (5.24 MB) are slightly higher. YOLOv8-RCAA has an mAP of 97.9% and an inference speed (45.6 FPS), yet its real-time performance on edge devices is still limited due to its 8.1G FLOPs and large model size of 6.46 MB. The mAP of the YOLO Tea model is 92.3%, but its inference speed (39.4 FPS) is low and model size (15.6 MB) is large, making it insufficient in real-time performance and under resource limitation condition of edge devices. The improved FCHE-YOLO model performs the best in terms of model size, detection speed, and computational complexity respectively, occupying a better overall performance, meeting UAVs’ requirements of precise detection of tea leaf diseases.

**Model Comparison and Innovation Analysis**

To further validate the innovation and advantages of FCHE-YOLO compared to existing advanced detection models of tea leaf diseases such as YOLOv8-RCAA, YOLO-Tea, RT-Detr, and ED-Swin Transformer[32], a detailed comparative analysis is conducted. Table 6 shows the differences between FCHE-YOLO and these four models in terms of backbone network structure, feature fusion structure, detection head structure, and inference efficiency.

| **Table 6 Comparative analysis of FCHE-YOLO and mainstream lightweight models** | | | | | | | |
| --- | --- | --- | --- | --- | --- | --- | --- |
| Model name | Backbone network structure | Feature fusion structure | Detection head structure | mAP_0.5 | Parameter count (M) | FLOPs (G) | Model characteristics and limitations |
| YOLOv8-RCAA | CSPDarknet + RepVGG | PANet + CBAM | Decoupled + ATSS | 97.9 | 5.6 | 5.2 | High accuracy, but low inference speed |
| YOLO-Tea | CSPDarknet53 + GCNet | RFB + ACmix | Common detection head | 92.3 | 15.6 | 7.9 | Good detection accuracy, yet high deployment cost |
| RT-Detr | Transformer Encoder-Decoder | FPN-like structure | Anchor-free Head | 93.4 | 90.3 | 21.2 | Complex large model structure with a poor real-time performance |
| ED-Swin Transformer | EMAGE module | DASPPmodule | Common detection head | 96.5 | 49.3 | 9.3 | Large model occupying strong multi-scale feature processing capability |
| FCHE-YOLO (in this paper) | FC_C3K2 (lightweight) | HSFPN (attention) | Efficient-Head（GConv） | 98.1 | 1.46 | 4.2 | High detection accuracy, best inference efficiency, suitable for edge deployment |

Table 6 shows the lightweight design and optimization of FCHE-YOLO on multiple key structures: With a self-designed FC_C3K2 module, the backbone network has fewer parameters and lower computational complexity; adopting HSFPN, the feature fusion part enhances the multi-scale feature expression ability combined with the attention mechanism; the detection head adopts an efficient Efficient-Head structure and introduces GConv to improve computational efficiency. FCHE-YOLO achieves the lowest parameter count (1.46 M) and lower FLOPs (4.2 G) while maintaining the highest detection accuracy (98.1%), significantly better than similar models, especially in resource-limited edge devices such as drones.

RT-DETR and ED-Swin Transformer perform well in terms of accuracy, but large parameter couont and low inference speed make them difficult to be effectively deployed in UAV devices. Considering the practical application scenarios i.e. UAVs and embedded devices, FCHE-YOLO via its efficient design can run better in resource-constrained environment while meeting detection accuracy requirement.

In summary, FCHE-YOLO balances detection accuracy, computational efficiency, and model lightweight in structural design, particularly suitable for deployment in edge devices such as UAVs and embedded systems, demonstrating better application potential.

•	The dataset used in this study consists of approximately 5,400 images obtained through data augmentation, all originally collected from a single tea plantation (Siwangshan). This limited source of data raises valid concerns regarding the potential for overfitting and questions the generalizability of the model to other locations and conditions. Moreover, The data augmentation techniques could be described with precise parameter ranges (e.g., how much brightness adjustment? what rotation angles?).

I suggest the authors to provide a thorough discussion of possible site-specific biases and clarified whether data from additional plantations, different seasons, or varying lighting conditions were included in the dataset or are planned for future work.

**Reply:** Thank you for your valuable suggestions! Since the tea garden where data was collected for this paper is located in Henan, and tea leaves typically grow in spring, the dataset only covers the spring season. In Section 2.2, the chapter on image acquisition, we have supplemented the description that the tea garden images were captured under different lighting conditions (such as sunny and cloudy days) and added relevant pictures for illustration. Additionally, in the outlook section, we mentioned that in the future, we will collect tea garden data from other regions (such as Anxi and Wuyishan) to verify the generalization ability of the model. Secondly, in terms of data augmentation, we employed random brightness adjustment and rotation angles. Specifically, the range of brightness adjustment is set to [-20%, +20%], and the rotation angle is randomly generated within the range of [0°, 360°], which we have explained in Section 3.1, the part concerning the tea leaf disease dataset.

**The detailed details are as follows:**

The images are also collected under different lighting conditions, including strong, normal, and weak light. The different sample images are shown in Figure 6.

(a) Strong light	(b) Normal light	(c) Weak light

Fig.6 Sample images of different lighting conditions

•	The comparative experiments presented in the manuscript primarily concentrate on conventional models such as Faster R-CNN, SSD, and earlier versions of the YOLO series. However, the recent advancements in transformer-based or hybrid CNN-transformer detection frameworks, such as DE-TR and Swin-Transformer, are not addressed or evaluated in this study.

I recommend the authors to discuss why transformer-based or attention-heavy detectors were not compared or tested. Even if not deployed on UAVs due to resource constraints, this would contextualize the chosen architecture.

**Reply:** Thank you for your valuable suggestions! Regarding your question about the comparison with Transformer and hybrid CNN-Transformer architectures (such as Swin-Transformer, DETR, etc.), we have added a comparative analysis of these representative Transformer methods in Section 4.4.3. In the discussion, we clearly point out that although these methods perform well in terms of accuracy, their large number of parameters and high inference latency make them difficult to deploy on UAV devices with limited resources. Therefore, considering that the main goal of this study is to improve the inference efficiency of the model on UAV devices, FCHE-YOLO was chosen as the main comparison object.

•	Although the model demonstrates high frame rates on a desktop GPU, its performance decreases to approximately 15 FPS when deployed on edge UAV devices, which could limit its real-time effectiveness in the field. While the manuscript briefly mentions plans for model pruning and TensorRT optimization, I recommend authors to include more specific details on current inference latency and energy consumption during UAV operations. Providing this information would offer a clearer understanding of the model’s practical feasibility and highlight any technical constraints that need to be addressed for successful real-world deployment.

**Reply:** Thank you for the reviewer's suggestions. To more comprehensively evaluate the actual deployment performance of the model on edge devices, we have added the measured results of FCHE-YOLO on the Jetson TX2 platform in the outlook section, including the average inference latency (approximately 66ms), frame rate (15.1 FPS), power consumption (approximately 14.3W), and video memory usage (approximately 1.2GB), as detailed in Table 7. Although the current version has not yet been accelerated using TensorRT, the results indicate that the model already has preliminary edge deployment feasibility. In the future, we will further improve the response speed and energy efficiency through model pruning and high-performance deployment optimization.

**The detailed details are as follows:**

At present, the detection speed of FCHE-YOLO model on desktop GPU devices can reach 47.5 frames per second, showing a good real-time detection capability. However, when deployed to edge devices such as Jetson TX2, the detection speed drops to 15.1 frames per second, equivalent to an average inference delay of 66 ms. Due to the limited computing resources of edge devices, real-time response capability faces certain challenges. To further analyze the feasibility of actual deployment, this paper further tests the performance of FCHE-YOLO on Jetson TX2, and the relevant indicators are shown in Table 7.

| **Table 7 Performance indicators of FCHE-YOLO on Jetson TX2 Platform** | | |
| --- | --- | --- |
| **i**ndicator | Value | Description |
| Inference delay | About 66 ms  approximately 66 ms | Average inference time per image |
| Detecting frame rate | 15.1 FPS | Tested frame rate, supporting low-speed real-time detection |
| Power consumption | About 4.3 W | Tested average power consumption (during operation) |
| Video memory usage | About 1.2 GB | GPU memory space required for stable operation |

Although currently the model has preliminary edge deployment capability, there is still room for further optimization. In the future, it is planned to improve the model and accelerate its detection through model pruning [33], and TensorRT [34] and quantization deployment methods. Additionally, regarding UAV shooting path planning, currently a simple rectangular flight path is employed, which works well in some tea gardens. The later plan is to optimize UAV shooting paths by leveraging the adaptability and efficiency of improved ant colony algorithms [35], which will enhance overall efficiency and ensure precise coverage of all areas that need to be detected. In terms of data collection, the data set will be expanded by collecting tea garden data from other regions (such as Anxi and Mount Wuyi), so as to verify the generalization ability of the model in different environments.

•	Many hyperparameters are listed, but key training details such as random seed, augmentation pipeline code, and training/validation split reproducibility are missing. I recommend authors to consider adding the full training pipeline and data splits to a supplementary file or public repository to meet PLOS ONE’s data availability standards.

**Reply:** Thank you for the reviewer's valuable suggestions. In the dataset section, we have explained that this study adopts an 8:1:1 splitting strategy for the training set, validation set, and test set. In addition, to ensure the reproducibility of the experimental results, we fixed the random seed at 42 during the training process to eliminate randomness between different experiments. To enhance the reproducibility of the experiments, we have uploaded the .yaml configuration file containing complete hyperparameter settings to the supplementary materials for readers' reference.

It should be noted that the current research is an ongoing project, and the overall system has not yet been accepted. It involves some key modules that have not been made public, so it is temporarily inconvenient to open-source all the source code. We promise that after the project is concluded, we will open part of the core code and model structure in a timely manner to further support academic exchanges. At the same time, we have provided as detailed configuration information as possible in the paper and supplementary files to meet the data availability policy requirements of PLOS ONE and ensure that others can understand and reproduce the main experimental procedures of this study.

**Minor Comments**

•	The manuscript has minor grammatical issues and redundant phrases. For example, “the model significantly improves the adaptability and expressiveness” could be shortened, in addition, some references are embedded mid- sentence and break the flow

I Recommend authors a careful language editing and to ensure consistent citation style;

**Reply:** Thank you for the reviewer's valuable suggestions. We have comprehensively polished the entire manuscript, unified the expression styles, deleted redundant phrases (e.g., revised "improve adaptability and expressive ability" to "enhance model robustness"), and standardized the format of references.

•	Figures illustrating the system architecture and modules should be labeled more clearly and be of higher resolution. Some figures (heatmaps, detection examples) are low-resolution and need clearer labeling.

I recommend to use high-resolution annotated figures with consistent color maps and scale bars.

**Reply:** Thank you for the reviewer's valuable suggestions. We have redrawn all the figures (Figure 1, Figure 19, Figure 20, etc.) in high definition, unified the color schemes and scales, and added explanatory notes for the abbreviations in the figures to ensure that the graphic information is complete and consistent.

•	Some cited papers (e.g., [20], YOLO-Tea) should be cross-checked for accuracy and updated if more recent works exist.

**Reply:** Thank you for the reviewer's valuable suggestions. We have cross-checked and cited the referenced literatures such as YOLO-Tea and YOLOv8-RCAA.

Reviewer #1

The abstract of the paper needs a revision. It needs to be precise and must convey the summary and not the details of each method.

**Reply:** Thank you for the reviewer's valuable suggestions. We have rewritten the abstract, focusing on the core content, refining the language, removing technical details, and retaining only the key innovations and main experimental results (see the abstract section).

The images are of low quality. They need to have clear labels, with words spelled out fully instead of just abbreviations.

**Reply:** Thank you for the reviewer's valuable suggestions. We have recreated some images in high-definition vector format, with complete labels in the legends. All abbreviations are accompanied by their full English names when they first appear in the main text.

The figures throughout the paper convey too less information. They need to be self-explanatory and elaborative.

**Reply:** Thank you for the reviewer's pointing out this issue. We have supplemented numerical explanations and comparative analyses at several key positions in the paper. Specifically, after Tables 4 and 5, we have added detailed interpretations of the changes in various indicator values, clearly analyzing the advantages of FCHE-YOLO in terms of mAP, F1, FPS, FLOPs, etc., as well as the reasons behind them. In the parts of Figures 17 and 18, we have further explained the trends of various indicators with training iterations and the balance of model performance reflected by the radar charts. These supplements help readers more clearly understand the logic of model improvement and the practical significance behind various figures. Please refer to the updated content in relevant sections such as Section 3.4 Experiments and Analysis and Section 3.5 Comparison of Detection Results.

You must provide full names before abbreviations throughout the paper.

**Reply:** We appreciate the reviewer's attention to the linguistic standardization of this paper. We have checked all abbreviations throughout the full text to ensure that their complete English names are provided when they first appear (e.g., Group Convolution (GConv), mean Average Precision (mAP), Frames Per Second (FPS), etc.), and their usage has been uniformly standardized. Abbreviations are no longer used independently in figure captions, table headers, or section titles to ensure accurate and clear expression.

The paper’s English can be improved. It is suggested to get it proof read.

**Reply:** Thank you for the reviewer's valuable suggestions. We have checked the English throughout the paper and also invited an English teacher to polish the entire text for us.

The organization of the paper can be improved and information shared systematically.

**Reply:** Thank you for the reviewer's valuable comments. We have merged Chapter 2 and Chapter 3 into "Materials and Methods" and adjusted the order of paragraphs to make the logic more compact.

•	The image quality of Figure 1 is poor. I was unable to read it even after downloading it. Also, the abbreviations need to be defined for a reader to understand the process. The backend system	also needs to be elaborated upon. This need to be observed for all the images.

**Reply:** Thank you for the reviewer's comments on image quality and information clarity. We have redrawn Figure 1, simplifying the original complex flow chart that contained a large number of network structure details into a clear system schematic. This new version highlights the overall process of UAV inspection and shooting, tea disease detection, and the background system, avoiding excessive redundant content that might interfere with readers' understanding.

**The detailed details are as follows:**

**Materials and Methods**

Materials and Methods Combining UAV remote sensing technology with an object detection model, this paper develops an automated tea leaf disease detection system. The system adopts UAVs to collect image data of tea gardens efficiently, and then employs the improved YOLO11 model to carry out the disease detection and localization, achieving real-time monitoring of tea diseases. The operational process is shown in Figure 1. The core function of the UAV system is to collect high-definition images through real-time aerial photography and ensure comprehensive coverage of all areas in the tea garden. Using the object detection model, image data is rapidly processed and analyzed to identify the types and regions of leaf diseases, facilitating quick and accurate prevention and control of leaf diseases.

To better support disease detection and decision-making, the system incorporates a backend management module. The backend system is capable of recording and displaying the disease category, location, and quantity for each detection area. Managers can view real-time disease data, generate statistical reports, and adjust prevention and control strategies based on the detection results through the backend. Furthermore, via data storage and query operations, the backend enables longitudinal tracking of disease incidence trends, thereby providing data support for the tea garden health management.

**Fig.1 Precise detection workflow for tea leaf diseases using UAVs**

•	In the hardware system, one of the core items are the cameras. Provide further explanation and use of multiple cameras.

**Reply:** Thank you for the valuable suggestions put forward by the reviewer. We have supplemented the detailed information of the camera and explained the reasons for using a single OAK-D depth camera as well as the feasibility of expanding it into a multi-camera system in the future.

The detailed content is as follows:

The system adopts the OAK-D depth camera as the core image acquisition device. Equipped with a binocular stereo camera and an RGB main camera, the OAK-D is capable of synchronously outputting depth maps and high-definition images, which can meet the dual requirements of target detection and disease area localization. This camera has the ability to capture 6DoF (six degrees of freedom) spatial information, which can enhance the geometric stability of images and improve robustness when shooting on uneven terrain. Currently, the system is deployed with a single OAK-D camera, considering the UAV's carrying capacity and cost control. However, the system supports horizontal expansion into a multi-camera array to improve coverage breadth and image redundancy, thus having good scalability in future field deployments.

•	What are your criteria, justification and reasoning of selecting this particular field and planning the path as defined? What are you referring to when you say UAV flew at 10-meter intervals? It is not clear.

**Reply:** Thank you for the reviewer's valuable suggestions. We have supplemented the explanations regarding the principles of tea garden path planning, as well as the meaning and basis of the 10-meter interval, so as to add the theoretical basis for the path design.

Specific revised content:

The experiment was conducted at the Siwangshan Tea Planting Base, with a total planting area of approximately 50,000 square meters. To ensure comprehensive coverage and appropriate overlap of images, the route design adopted parallel equidistant path planning, as shown in Figure 3. The UAV flies at an equidistant interval of 10 meters, which, combined with its visual coverage range, ensures a high overlap rate and blind-spot-free shooting of the entire planting area, thus obtaining comprehensive high-definition image data. The so-called "10-meter interval" refers to the lateral distance between adjacent routes, which is comprehensively determined based on the camera's field of view (approximately 120°), flight altitude (10–15 meters), and image overlap requirement (>80%). The UAV flies at such equidistant intervals to ensure that each area is captured at least twice, avoiding image blind spots and omissions.

**Fig.3 UAV flight path**

•	It is not clear to me if the drone was custom built or off the shelf?

**Reply:** Thank you for the reviewer's valuable suggestions. The DJI T25P agricultural UAV used in this paper is an off-the-shelf commercial model, featuring high load capacity, long endurance, obstacle-avoidance flight, and other capabilities, which makes it suitable for aerial photography missions over large-area farmland. On this basis, to meet the requirements of real-time disease detection tasks, we have expanded the functions of this UAV system by additionally integrating an AI computing box (Jetson TX2) as the edge inference platform, along with an external high-resolution OAK-D camera and a GPS module. This enables functions such as image acquisition, target detection, and fusion of position information. Therefore, the overall UAV platform adopts a design concept of "off-the-shelf hardware + custom-mounted modules" to balance system stability and flexibility in algorithm deployment.

•	What was the purpose and use of other sensors in your study as you have defined in your DJI selected drone, Table 1.

**Reply:** Thank you for the reviewer's valuable suggestions. We have supplemented explanations regarding the functions of various sensors (such as GPS, obstacle avoidance sensors, radar, etc.) and their usage in this study. In addition to the camera, the UAV platform is also integrated with a GPS module, a downward-looking optical flow sensor, front and rear obstacle avoidance sensors, and a phased array radar: - The GPS module is used for accurately positioning the UAV's flight path, ensuring a high degree of consistency with the ground route planning.- The downward-looking optical flow sensor serves to maintain the stability of low-altitude flight, providing compensation especially in areas with weak GPS signals.- The obstacle avoidance sensors and radar enhance flight safety by automatically evading sudden obstacles, such as protruding tree crowns or abrupt terrain changes.

•	The drone seems to be significantly large with 26kg weight. How this aligns with your claim of compact size drone. These two seem to be contradictory.

**Reply:** Thank you for pointing out the imprecision in the wording. We agree that the term "compact" is not accurate here and may cause misunderstanding, so we have deleted this description in the paper and uniformly replaced it with "medium-sized agricultural UAV". The DJI T25P used in this study is an off-the-shelf commercial agricultural UAV with an overall weight of 26kg, and its design goal is to support high-load operations. In addition to being used for image acquisition and disease detection, this platform also reserves space and load capacity for carrying a spraying system, facilitating the subsequent realization of functions such as automatic pesticide spraying. Therefore, the weight of 26kg is reasonable and necessary in the system design and future expansion of this study. We have further supplemented relevant explanations in the main text. Thank you for helping us improve the rigor of expression.

•	What was the height of your drone flight? It has not even been represented in your Figure 4.

**Reply:** Thank you for the reviewer's valuable suggestions. We have supplemented the information on flight altitude and annotated this information in the caption of Figure 4.

The specific revised content is as follows:

The so-called "10-meter interval" refers to the lateral distance between adjacent flight routes, which is collectively determined by the field of view (about 120°), flight altitude (10-15 m), and image overlap requirement (>80%). The UAV flies equidistant along this path, ensuring that each area can be captured at least twice to prevent imaging blind spots and coverage gaps.

In actual data collection, the flight altitude of the drone is set to 10-15 m, which can maintain a stable perspective and image resolution in both flat and sloping environments. This height is the optimal value determined after comprehensively considering the camera resolution (4032 × 3024), crop leaf size, and detection algorithm performance. The flight attitude of the drone is shown in Figure 4, where H is 10 m and R-angle is 60°.

**Fig.4 UAV body attitude during photography**

•	How frequently images were captured and what was the reasoning behind this interval?

**Reply:** Thank you for the reviewer's valuable suggestions. We have clarified the frequency of image capture and the basis for its design.

The specific revised content is as follows:

To improve the adaptability of the detection system to changes in external conditions, image acquisition is carried out in different time periods (8:00-11:00, 14:00-17:00) and lighting conditions. The shooting frequency is set to 1 frame per second (1 fps), corresponding to a flight speed of 4-5 m/s approximately. This frequency, which ensures image overlap while reducing redundant images and post-processing burden, is a compromise between image density and system efficiency.

•	I would suggest to create a table and define the key parameters of your flight and planning.

**Reply:** Thank you for the reviewer's valuable suggestions. Following the recommendations, we have added Table 1 to systematically summarize the key parameters of flight and image acquisition.

The specific table is as follows:

**Table 1 DJI T25P UAV Aerial Photography Parameters**

| Parameter category | Parameter name | Value/range | Specification |
| --- | --- | --- | --- |
| Flight parameter | Flight altitude | 20-25 m | Balance field of view coverage and clarity |
|  | Flight line spacing | 10 m | Horizontal spacing between adjacent flight routes |
|  | Flight speed | 4-5 m/s | Control the overlap density of images |
|  | Image collection time period | 8:00-11:00，14:00-17:00 | Adapt to different lighting conditions |
| Camera  parameter | Image resolution | 4032×3024 px | Ensure clear blade characteristics |
|  | Shooting frequency | 1 fps | Control data volume and coverage density |
|  | Image format | JPG | Balance compression and image quality |
| Sensor | GPS module | High precision positioning | Ensure accuracy of path reproduction and data annotation |
|  | Optical flow/obstacle avoidance sensor | Real-time stable flight and safety assurance | Assisted navigation and obstacle avoidance |

•	In the start of your paper, you have stated mainly two types of tea plant diseases, but then in

your image selection section, you have stated more than 8 type of diseases. This makes the

study a lot more complex. You must state clearly what is the scope of your study in the start and stick to the plan instead of taking into account additional factors for your study to be focused.

**Reply:** Thank you for the reviewer's attention to the consistency of the research content. In the initial draft, we focused on introducing two representative tea plant diseases at the beginning, aiming to present the research background and practical needs. However, during actual image collection, considering the project acceptance and application scenarios, we randomly collected a large number of naturally distributed disease samples, and finally constructed an image dataset containing 8 common disease types to improve the generalization ability and practicality of the model. To ensure the clarity and consistency of the research objectives, we have supplemented in the introduction that the research objects cover 8 major tea plant diseases such as leaf rust and brown blight, making it clear that this study aims to achieve unified detection and identification of multiple diseases. The relevant descriptions have been revised simultaneously to avoid readers' doubts about the inconsistency of the scope.

•	Why 3,000 images were captured, why this number.

**Reply:** Thank you for the reviewer's valuable suggestions. The reason for capturing 3,000 photos is to ensure that each category has an adequate number of samples for model training. With 8 categories in total, it is essential to guarantee sufficient data volume for each category to avoid the model being biased toward certain categories. 3,000 photos is a reasonable number, as it provides a balanced sample size for each category, enabling the model to learn the characteristics of every category. In addition, this number also takes data diversity into account, ensuring that the model can cope with different scenarios and environmental changes, thereby improving the accuracy and robustness of classification. Of course, we may continue to increase the number of original images in the future to further enhance the model's performance and generalization ability.

**Methods**

•	Explain the role of every method you have used and align it with your research questions and also the function you are achieving in a summary table to show how these different methods come together to achieve an overall outcome.

**Reply:** Thank you for the reviewer's valuable suggestions. We have added detailed explanations of each method in the conclusion section of the paper, integrating them with the research questions and objectives. We have specifically elaborated on how each method (such as the optimization module of the FCHE-YOLO model) addresses issues in existing technologies, including complex backgrounds, insufficient detection accuracy, and excessive computational load. Additionally, we have explained how these methods work synergistically to enhance the overall performance.

The specific revised content is as follows:

Conclusions

Tea leaf diseases have a significant effect on the yield and quality of tea. High-precision automatic detection and identification of tea leaf diseases are beneficial for precise disease prevention and control. This study proposes an improved YOLO11 model, called FCHE-YOLO, to improve the accuracy and real-time capability of tea leaf disease detection, and embeds it into UAV devices to meet practical application demands.

To address the low accuracy, excessive computational load, and overfitting of current detection methods in complex backgrounds, multiple optimizations are conducted based on YOLO11:

① FC_C3K2 module: In the backbone network, a new lightweight module FC_C3K2 is introduced, which combines FasterNet Block, Convolutional Gated Linear Unit (CGLU), and C3K2 structure. This module can significantly reduce the parameter count and computational complexity, and enhance the robustness of the model in complex background, especially with good adaptability on edge devices. This optimization addresses the accuracy shortcomings of existing technologies when handling complex backgrounds.

② HSFPN module: In the neck network, the High-level Screening Feature Pyramid Network (HSFPN) is adopted to replace the traditional feature pyramid network. HSFPN enhances the multi-scale detection capability and robustness of the model by efficiently integrating features of different levels, while effectively reducing the model’s storage and computational load. This optimization not only enhances the model accuracy, but also makes the improved YOLO11 lighter, more efficient, and particularly suitable for edge device deployment.

③ Efficient-Head module: In the detection head section, an Efficient-Head structure is designed. It uses Group Convolution (GConv) instead of traditional convolution, thereby reducing the training parameters and computational complexity of the model. This module effectively suppresses overfitting of the model while improving detection accuracy, ensuring good performance on resource-constrained devices.

The results based on the self built dataset from UAVs show that FCHE-YOLO improves the average recognition accuracy (mAP) by 4%, and recognition speed (FPS) by 9%, and reduces the floating-point operations (FLOPs) by 34.3%, parameter count by 38.9%, and memory size by 41.9% compared to the YOLO11 model. Hence, the FCHE-YOLO model not only has a higher accuracy and a faster speed, but also effectively reduces the missed detection in complex backgrounds.

Through these innovative optimizations, FCHE-YOLO demonstrates its advantages in edge computing devices (such as UAVs), delivering an efficient and deployable solution for tea leaf disease detection. These findings provide important references for the future application of object detection methods based on deep learning in agricultural monitoring.

•	The network structure you have presented in Figure 6 for YOLO is already quite complicated for a general reader. You must provide a high-level overview with clear names and labels before

diving into a detailed structure.

**Reply:** Thank you for the reviewer's valuable suggestions. In accordance with the reviewer's recommendations, we have added a concise overview before Figure 6, which explains the high-level framework of the YOLO network structure and briefly introduces the functions of the backbone network, neck network, and detection head. Subsequently, in Figure 6, we have shown the specific structure of YOLO11 in detail and explained the optimization and role of each module, enabling readers to better understand the structure.

The specific revised content is as follows:

**YOLO11 Network Structure**

YOLO11 is the latest version in the YOLO series, optimized from YOLOv8 to achieve 171 better performance on the COCO dataset with fewer parameters. In the YOLO11, the backbone and neck networks are both redesigned, and a new module called C3K2 is introduced, which significantly enhances the model’s feature extraction capability, enabling itself to perform better in multi-object detection tasks. YOLO11 comprises 3 core modules: backbone network, feature fusion layer, and detection head, the network structure of which is shown in Figure 7.

**Fig.7 YOLO11 model structure diagram**

Backbone is the fundamental component of YOLO11, responsible for extracting multi-scale features from input images. The composition of the backbone network includes multiple key modules, ensuring that the model can efficiently extract local and global features. Firstly, the Conv (Conv Layer) module is the initial part of the backbone network, responsible for basic low-level feature extraction of input images, providing a foundation for subsequent high-level feature extraction. Next, the C3K2 module integrates multiple convolutional layers and bypass connections, and uses flexible convolutional configurations and bottleneck designs to improve feature extraction capability. By introducing the Cross Stage Partial (CSP) architecture, C3K2 optimizes gradient flow and enhances local feature extraction, improves training stability, and enhances the robustness of YOLO11 in complex backgrounds. Finally, the SPPF (Spatial Pyramid Pooling Fast) module enhances the model's ability to capture global contextual information through multi-scale pooling operations, while the C2PSA (Cross-stage Partial Spatial Attention) module improves the model's detection ability in complex scenes by effectively filtering irrelevant information and emphasizing key features, showing significant advantages especially in multi-target detection and occlusion problems.

The Neck module connects Backbone and Detection Head, responsible for the fusion of multi-scale features, particularly suitable for detecting objects of different sizes. Its core task is to enhance object detection performance and ensure that the model can maintain efficient performance on multiple scales. Firstly, the Upsample module helps the model better detect smaller objects by increasing the resolution of low-resolution feature maps. Secondly, the Concat (series connection of features) module fuses feature maps with different resolutions, enabling the model to utilize multi-scale information to improve detection accuracy. Finally, the C3K2 module enhances feature representation capability and improves detection accuracy in Neck by performing repeated convolution operations and quick connections of multi-scale fuse features. The introduction of the C3K2 module enables the model to achieve stronger expression ability on different scales, thereby improving overall detection performance.

The Detection Head is the final stage of YOLO11, responsible for converting processed feature maps into accurate object detection results. With the multi-scale capability, it ensures excellent performance of the model in object detection tasks of various sizes. Firstly, the classification detection head adopts the depthwise separable convolution (DWConv) structure, which is a lightweight convolution method that improves computational efficiency by simplifying convolution operations. DWConv reduces computational cost while maintaining high detection accuracy through a combination of deep convolution and pointwise convolution. Next, the bounding box detection path captures accurate spatial information through standard convolution, ensuring precise prediction of the target boundary. Finally, the classification detection path utilizes DWConv for classification tasks, reducing computational complexity, and is particularly suitable for resource constrained environment such as edge device or embedded system. DWConv significantly reduces computational cost while ensuring accuracy, enabling YOLO11 to efficiently handle multi-objective scenes.

•	You are using abbreviations in heading which should usually not be the case. Abbreviations must be used in the text body and not the headings unless they are already defined.

**Reply:** Thank you for the reviewer's valuable suggestions. We have revised the phrase "this paper improves the FCHE-YOLO model" in the title to "the improved YOLO11 model", and corresponding adjustments have also been made to the titles of other sections.

•	Within your proposed structure in figure 7, you must explain the logic behind the presence of repeated FasterNet Blocks.

**Reply:** Thank you for pointing out this issue. To enhance the network's robustness in complex backgrounds and reduce computational overhead, repeated FasterNet Blocks are used in Figure 7. Each block is responsible for different feature extraction and optimization tasks. By introducing multiple FasterNet Blocks, we can improve the network's adaptability to complex backgrounds while further enhancing detection accuracy while maintaining lightweight properties. We will add this explanation in the paper and further elaborate on its specific contribution to network performance in the experimental section.

The specific revised content is as follows:

**FasterNet Block Structure**

FasterNet [24] is an efficient neural network architecture that employs the innovative Partial Convolution (PConv) technique. PConv reduces computation and memory access by processing only a portion of the input channels, as shown in Figure 7. FasterNet is a four-layer hierarchical architecture, with each layer composed of a series of FasterNet Blocks and supplemented with embedding or merging operation layers.Within each FasterNet Block, the core component is the PConv layer, subsequently enhanced by two regular convolution (Conv) layers. In the FasterNet architecture, FasterNet Blocks are repeatedly used to enhance the network's feature extraction capability. Each block is responsible for extracting features from different levels, by which the network is capable of handling more complex and dynamically changing inputs without significantly increasing computational cost. Reusing FasterNet Block blocks has several advantages as follows:

193

① Improving robustness: Each FasterNet Block extracts features from different scale and level, which can effectively enhance the model's adaptability to complex background.

② Optimizing computing resources: Due to that PConv reduces the computational load and memory access of each convolutional layer, reusing these blocks not only avoids redundant computing, but also improves the inference efficiency of the network on edge devices.

③ Reduce computational redundancy: By reusing the FasterNet Block, FasterNet can improve the model's ability to capture details and express features without increasing excessive computing resources.

**Experiments and Analysis**

•	Authors mentioned section 3.2 but where is it in the paper?

**Reply:** Thank you for the reviewer's valuable suggestions. We have checked and revised Section 3.2 on the experimental environment to ensure its correct positioning in the paper. The numbers of relevant paragraphs and sections have been adjusted to ensure consistency with the content of the paper.

•	Results in Table 4 must highlight the top three models. Label your model as (Our) in the table along with the name.

**Reply:** Thank you for the reviewer's valuable suggestions. In Table 4, we have clearly marked the top three models and used bold formatting to highlight the results of our model. We have also attached a clear name to the improved model in this paper, ensuring that readers can easily see the performance differences between our improved model and other comparative models.

The specific table is as follows:

**Table 4. Performance Comparison of Optimization Module Ablation Experiments**

| Model | FasterNet | FC  C3K2 | HSFPN | Efficient  -Head | P/% | R/% | mAP  0.5/% | FPS  (f/s) | FLOPs  /G | Parameters  /M | Size  (MB) |
| --- | --- | --- | --- | --- | --- | --- | --- | --- | --- | --- | --- |
| YOLO11 | × | × | × | × | 95.3 | 93.4 | 94.1 | 43.3 | 6.4 | 2.59 | 5.24 |
| YOLO11-F | √ | × | × | × | 95.8 | 94.1 | 94.6 | 44.2 | 5.9 | 2.30 | 4.67 |
| YOLO11-FC | × | √ | × | × | 96.5 | 94.6 | 95.3 | 43.5 | 5.7 | 2.24 | 4.57 |
| YOLO11-H | × | × | √ | × | 97.5 | 95.1 | 96.2 | 44.8 | 5.7 | 1.83 | 3.76 |
| YOLO11-E | × | × | × | √ | 96.2 | 94.5 | 95.1 | 43.5 | 5.2 | 2.32 | 4.70 |
| YOLO11-FCH | × | √ | √ | × | 97.9 | 97.2 | 97.5 | 44.9 | 5.3 | 1.66 | 3.44 |
| FCHE-YOLO | × | √ | √ | √ | 98.9 | 97.8 | 98.1 | 47.5 | 4.2 | 1.46 | 3.04 |

•	Provide F1 score results for the combined precision and Recall.

**Reply:** Thank you for the reviewer's valuable suggestions. To evaluate the model's performance more comprehensively, we have added the F1 score to the table. The F1 score is the harmonic mean of Precision and Recall, which can better reflect the model's performance on imbalanced datasets. The mAP (mean Average Precision) in the table represents the mean average precision, serving as a traditional evaluation metric. The newly added F1 score, on the other hand, provides a more comprehensive performance measure, helping to more accurately understand the model's effect in practical applications.

•	What was the basis of selecting various models for comparison. Full justification nee dto be provided.

**Reply:** Thank you for your valuable comments. We have selected a variety of mainstream object detection models for comparison, mainly based on the following reasons: First, these models perform excellently in the field of computer vision and have strong representativeness; second, the selected models involve different network architectures (such as YOLO series, Faster R-CNN, Transformer models, etc.), which can fully demonstrate the advantages of our proposed FCHE-YOLO model over other architectures. We will supplement these selection criteria in the paper to ensure the rationality of the comparison.

The detailed details are as follows:

**Comparison of Algorithm Performance**

To verify the detection performance of the proposed FCHE-YOLO model, several mainstream object detection models are selected for comparative experiments, which cover different types of network architectures, including YOLO series (v5n, v8n, v10n, v11n), Faster R-CNN, SSD, Transformer structure RT-DETR series models, as well as recently proposed improved versions such as YOLO Tea and YOLOv8-RCAA.The reasons for choosing these models are multifaceted.

Firstly, these models have a wide range of applications in the field of computer vision and perform excellently in their respective domains. YOLO series and Faster R-CNN models are classic models in the object detection field, and their excellent performance is proven in multiple practical tasks. Therefore, choosing these models as comparison objects is to fully verify the advantages of the FCHE-YOLO model. Secondly, the comparison with these models occupying different architecture and characteristics benefits to demonstrate the advantages of FCHE-YOLO from multiple dimensions. The YOLO series models are mainly single-stage detection networks, emphasizing real-time performance and inference speed, especially suitable for edge device applications requiring efficient processing; Faster R-CNN is a two-stage detection network that performs well in accuracy, but has slow inference speed and high computational cost; the RT-DETR model adopts the Transformer architecture and exhibits certain advantages in handling complex target relationships, but large computational load makes it unsuitable for running on resource constrained devices. YOLO-Tea and YOLOv8-RCAA as improved models for tea disease detection are incorporated, providing a comparative perspective to validate the advantages of FCHE-YOLO in this specific task.

The above models covering different types of architectures can verify the performance advantages of FCHE-YOLO by comparison from detection accuracy, model complexity, and deployment efficiency. Table 5 presents the comparative results of different object detection models.

**Table 5. Performance Comparison of Different Detection Models**

| Model | P/% | R/% | F1/% | mAP0.5/% | FPS(f/s) | FLOPs/G | Size(MB) |
| --- | --- | --- | --- | --- | --- | --- | --- |
| Faster RCNN | 79.5 | 78.3 | 73.5 | 78.6 | 6.5 | 209 | 149 |
| SSD | 68.1 | 67.3 | 64.7 | 67.7 | 28 | 29.5 | 90.1 |
| YOLOv5n | 89.5 | 86.1 | 85.1 | 88.4 | 35.6 | 6.3 | 14.7 |
| YOLOv8n | 91.5 | 90.6 | 88.9 | 91.2 | 41.3 | 8.9 | 6.23 |
| YOLOv10 | 89.5 | 90.0 | 86.4 | 89.7 | 39.5 | 6.7 | 5.6 |
| RT-Detr | 93.7 | 93.1 | 90.6 | 93.4 | 37.1 | 21.2 | 63.4 |
| YOLO11 | 95.3 | 93.4 | 92.2 | 94.1 | 43.3 | 6.4 | 5.24 |
| YOLOv8-RCAA | 98.2 | 85.3 | 91.3 | 97.9 | 45.6 | 8.1 | 6.46 |
| YOLO-Tea | - | - | **90.4** | 92.3 | 39.4 | 7.9 | 15.6 |
| FCHE-YOLO | **98.9** | **97.8** | **96.0** | **98.1** | **47.5** | **4.2** | **3.04** |

The experimental results show that the improved FCHE-YOLO model performs optimally overall, with mAP 0.5 reaching 98.1%. Compared to Faster R-CNN, SSD, YOLOv5n, YOLOv8n, YOLOv10, RT-Detr, YOLO11, YOLOv8-RCAA and YOLO-Tea models, it shows the mAP is improved by 19.5%, 30.5%, 9.7%, 6.9%, 8.4%, 4.7%, 4%, 0.2% and 5.8% respectively, and the F1 score is increased by 22.5%, 31.3%, 10.9%, 7.1%, 9.6%, 5.4%, 3.8%, 4.7%, and 5.6% respectively.

Specifically, Faster R-CNN has a floating-point operation count of 209 G with a large model size and slow detection speed, failing to meet real-time detection requirements. The single-stage detection model SSD, with a weight of 90.1 MB and detection speed of 29.5 frames per second, struggles to meet the real-time and resource-constrained requirements of UAV devices. Although the RT-Detr model exhibits excellent accuracy, its excessive parameter count makes it unsuitable for real-time detection on edge devices. Compared to the YOLO series algorithms, the improved FCHE-YOLO model performs best in model size, detection speed, and computational complexity, making its overall performance superior. The improved FCHE-YOLO model can meet the requirements for precise detection of tea leaf diseases by UAVs.

471

474

475

476

477

In the YOLO series algorithms, YOLOv5n performs well in speed (35.6 FPS), but its accuracy is relatively low, with a mAP of only 88.4%, inferior to FCHE-YOLO. YOLOv8n has a higher accuracy (mAP of 91.2%), a smaller model size (6.23 MB), and a good inference speed (41.3 FPS), yet there is a gap in accuracy and computational complexity compared to FCHE-YOLO. YOLOv10 achieves a good balance between accuracy (mAP of 89.7%) and speed (39.5 FPS), but still falls short of FCHE-YOLO's performance. YOLO11’s accuracy and inference speed improve to 94.1% and 43.3 FPS, respectively, close to FCHE-YOLO, however its computational complexity (6.4 G FLOPs) and model size (5.24 MB) are slightly higher. YOLOv8-RCAA has an mAP of 97.9% and an inference speed (45.6 FPS), yet its real-time performance on edge devices is still limited due to its 8.1G FLOPs and large model size of 6.46 MB. The mAP of the YOLO Tea model is 92.3%, but its inference speed (39.4 FPS) is low and model size (15.6 MB) is large, making it insufficient in real-time performance and under resource limitation condition of edge devices. The improved FCHE-YOLO model performs the best in terms of model size, detection speed, and computational complexity respectively, occupying a better overall performance, meeting UAVs’ requirements of precise detection of tea leaf diseases.

•	Radar Figure 18 must be further elaborated upon.

**Reply:** Thank you for the valuable suggestions on Figure 18. We will add a detailed explanation of the radar chart in the paper, specifically illustrating the evaluation metrics represented by each dimension, as well as the differences in performance between FCHE-YOLO and other models in each metric. We will further analyze why FCHE-YOLO performs excellently in multiple dimensions and explain how the results shown in the radar chart support the effectiveness of our proposed model.

Specific revised content:

To intuitively display the comprehensive performance of different models, this paper compares the models from Table 5 across multiple evaluation metrics, including precision (P), recall (R), mAP 0.5, average inference time, arameter count, and model size, visualized through a radar chart as shown in Figure 19. In the radar chart,each curve represents a model, with points closer to the edge indicating better performance on the corresponding metric; a larger enclosed area represents a stronger comprehensive performance.

**Fig.19 Multi-model performance comparison radar chart**

From Figure 18, although some models (such as RT-Detr series and YOLOv8 series) achieve good results in mAP 0.5, the FCHE-YOLO model demonstrates more prominent comprehensive performance, especially with clear advantages in the number of model parameters and detection speed. These advantages are crucial for model deployment and real-time requirements in practical applications, giving FCHE-YOLO an edge in practical scenarios.

From Figure 19, it can be seen that FCHE-YOLO exhibits a relatively balanced performance across all dimensions. Especially in the precision (mAP_0.5), F1 score (F1), precision (P), and recall (R), its curves are close to the edge of the radar chart, showing an excellent performance in detection accuracy and category discrimination. FCHE-YOLO can identify the targets of different categories accurately and effectively, and reduce missed and false detections.

In computational complexity and model volume, the FCHE-YOLO curve is closest to the center of the graph, occupying a lowest computational cost and a smallest model volume. These features make it very suitable for deployment in resource-constrained environment, especially when conducting real-time detection on edge devices such as drones, it can effectively reduce computational burden and improve inference speed.

As for RT-DETR, its accuracy is close to FCHE-YOLO and its performance is also good in mAP and F1 scores, but it has a large parameter count and a high FLOPs of up to 21.2 G, far higher than FCHE-YOLO (4.2 G), unsuitable for deployment on edge devices with limited computing power. YOLOv5n and YOLO-Tea have smaller model sizes and lower computational costs, but their detection accuracy and recall are significantly lower than FCHE-YOLO, especially in complex backgrounds false or missed detection are prone to occur.

Through comparison, the radar chart further confirms the comprehensive advantages of FCHE-YOLO in accuracy, real-time performance, and deployment efficiency, which are crucial for model deployment, real-time requirement, and adaptability to resource-constrained devices in practical applications. Therefore, FCHE-YOLO, occupying more advantageous in tea leaf disease detection, can provide efficient and high-precision detection results, especially on edge devices such as UAVs.

•	You have not provided any experimental conditions for UAV testing, and type of tests conducted + how many tests.

**Reply:** Thank you for your valuable comments. Detailed information regarding the experimental conditions, test types, and test quantities of the UAV tests has been described in Sections 2.2 and 3.1 of the paper. For the reviewer's convenience, the following is a brief supplement to the relevant content:

**Experimental location:** Siwangshan Tea Planting Base, with a total planting area of approximately 50,000 square meters. Image acquisition was conducted using a DJI T25P UAV, with a flight altitude of 10–15 meters and a route spacing of 10 meters, ensuring comprehensive coverage and appropriate overlap of images.

**Test types:** We performed target detection using images of tea leaf diseases captured by the UAV, covering multiple disease types such as tea black rot, tea brown blight, and tea leaf rust.

**Test quantity:** The total dataset of over 5,400 images was divided in an 8:1:1 ratio, with 4,328 images used for training, 541 for validation, and 540 for testing. Therefore, the final number of test images is 540.

Detailed content of this part has been further elaborated in Sections 2.2 and 3.1 of the paper. Thank you for your attention to our work.

•	Why only heat maps of two models were selected. Heatmap of all the comparative models in your study should be compared and discussed.

**Reply:** Thank you for your valuable comments. Regarding the selection of heatmaps, initially, only the heatmaps of the YOLO11 and FCHE-YOLO models were presented, mainly based on the significant differences in their performance. To fully demonstrate the effect of model optimization and in response to the reviewer's suggestions, we will add heatmaps of more comparative models in the revised version, including YOLOv5n, YOLOv8n, YOLO-Tea, etc. This will help comprehensively show the detection ability and robustness of different models in complex backgrounds, further verifying the advantages of our model.

Specific revised content:

To further intuitively demonstrate the detection effects of different models, heatmap processing was applied to the original image in Figure 22, with results shown in Figures 23-32. These figures display the heatmap results of OLO11 and FCHE-YOLO models, 503 intuitively presenting the possibility of model target detection through temperature changes.

Fig.22 Original image

Fig.23 Faster RCNN heatmap

Fig.24 SSD heatmap

Fig.25 YOLOv5n heatmap

Fig.26 YOLOv8n heatmap

Fig.27 YOLOv10 heatmap

Fig.28 RT-Detr heatmap

Fig.29 YOLO11 heatmap

Fig.30 YOLOv8-RCAA heatmap

Fig.31 YOLO-Tea heatmap

Fig.32 FCHE-YOLO heatmap

It can be seen that the heat maps of Faster RCNN, SSD, YOLOv5n, YOLOv8n, and YOLOv10n models are relatively scattered, in which environment and targets are difficult to be distinguished effectively, and targets are not focused accurately. Benefiting from the Transformer based architecture, RT-Detr has a strong focusing ability, which yet leads to the omission of small objects around the target. YOLO11 is difficult to distinguish between targets and backgrounds and identify target locations accurately. Especially in complex backgrounds, false or missed detection is prone to occur, easily misidentifying similar background objects as the target or failing to detect real target. The heatmaps of YOLOv8-RCAA and YOLO-Tea have a certain sensitivity to the target, but missed detection still exists. In contrast, FCHE-YOLO is more sensitive to targets and less affected by environmental interference. Although it cannot completely avoid background interference, FCHE-YOLO can more accurately identify and focus on target objects in complex background situations, and significantly enhances the ability to recognize targets in similar backgrounds.
