# Supplementary material for: Precise tea leaf disease detection using UAV low-altitude remote sensing and optimized YOLO11 model
Source: PLoS One. 2026 Feb 18;21(2):e0342545. doi: 10.1371/journal.pone.0342545 (PMC12915959; doi:10.1371/journal.pone.0342545)
Supplement: Attachment — Submitted filename: Response 2025.11.17.docx [file pone.0342545.s005.docx]

**Response to Reviewers’ Comments**

We sincerely thank the anonymous reviewers for their valuable comments and constructive suggestions on our manuscript. These comments are highly appreciated and have been of great help in revising and improving the paper. They also provide important guidance for our research. After carefully considering all the reviewers’ remarks, we have made substantial revisions to the manuscript and hope that it can now be accepted for publication.

In the revised manuscript, our responses to the reviewers’ comments are marked in red. Below is a detailed explanation of how we addressed each comment and suggestion in the updated version of the paper.

1.The remaining points are minor refinements that could further strengthen the paper: (i) briefly reiterate dataset limitations in the Conclusions for balance,(ii) include approximate energy consumption data from UAV deployment to reinforce field feasibility, and (iii) ensure the data and code repository fully complies with PLOS ONE standards, with unrestricted access to raw data, annotations, augmentation scripts, and trained weights.

Author Response：

(i) Briefly restate the limitations of the dataset in the conclusion section to maintain balance.

Response: Thank you very much for your valuable suggestion! We have added a discussion on the limitations of the dataset in the outlook section. Specifically, we note that the current data were mainly collected from the Siwangshan tea plantation in Xinyang, Henan Province, which exhibits strong regional characteristics and may limit the model’s generalization ability on tea plants from other regions. In addition, the amount of collected data is still relatively limited. In future work, we will further expand the sample size to enhance the robustness and generalization performance of the model.

The revised text is as follows:

It should be noted that the data used in this paper is mainly sourced from the Siwangshan Tea Plantation in Xinyang, Henan Province. The data has strong regional characteristics, which may lead to limited generalization ability of the model in other regions or on different tea varieties. In addition, the number of currently collected samples is still relatively limited. In the future, the dataset will be further expanded on this basis, and tea plantation data from other regions (such as Anxi, Wuyishan, etc.) will be collected to verify the robustness and generalization ability of the model in different environments.

(ii) Thank you for your valuable suggestions! In the Outlook section, we have supplemented the energy consumption description of the UAV equipped with the Jetson TX2 platform during field detection. The experimental results show that in the no-load state (without operation load): the power consumption is approximately 450–500 W, the energy consumption per operation is about 0.20 kWh, and the endurance is about 1.7 hours (102 minutes); in the full-load state (with a 20 kg load for spraying operations): the power consumption is approximately 600–650 W, the energy consumption per operation is about 0.26 kWh, and the theoretical endurance is about 1.3 hours (78 minutes). This supplement helps to verify the practical application feasibility of the system in tea garden scenarios.

The revised text is as follows:

Table 8. Operating Performance Indicators of FCHE-YOLO on the Jetson TX2 Platform

| Indicator | Value | Description |
| --- | --- | --- |
| Inference delay | About 66 ms  approximately 66 ms | Average inference time per image |
| Detecting frame rate | 15.1 FPS | Tested frame rate, supporting low-speed real-time detection |
| Power consumption | About 4.3 W | Tested average power consumption  (during operation) |
| Video memory usage | About 1.2 GB | GPU memory space required for stable  operation |

When the UAV equipped with Jetson TX2 performs field detection and spraying operations in tea plantations, the overall power consumption of the system varies with the operation status: in the no-load state (without operation load), the power consumption is approximately 450-500 W, the energy consumption per operation is about 0.20 kWh, and the theoretical endurance is around 1.7 hours (102 minutes); in the full-load state (with a 20 kg load for spraying operations), the power consumption is approximately 600-650 W, the energy consumption per operation is about 0.26 kWh, and the theoretical endurance is around 1.3 hours (78 minutes). The above-mentioned power consumption includes that of the flight motor, spraying/seeding pump, front and rear radars, and edge computing module.

(iii) Thank you for your valuable suggestions! We have carefully proofread the entire paper, eliminated redundant expressions, optimized sentence structures and paragraph transitions, which has made the paper’s logic more coherent and overall readability improved.

2.In the start of the section, only a brief intro of the backend system will be sufficient. I would suggest to include the detailed discussion on the backend system within the appropriate section, such as by introducing the software system components section. This relates to reorganisation of the paper.

Author Response：

Thank you for your valuable suggestions! Currently, the backend of our system is still in the development and testing phase, and the backend management module has not been fully implemented, so we cannot provide a complete demonstration or detailed technical description. Therefore, only a brief introduction to the backend system is given in this section of the paper to ensure the integrity of the paper's structure.

The revised text is as follows:

To better support disease detection and decision-making, the system incorporates a backend management module. The backend system is capable of recording and displaying the disease category, location, and quantity for each detection area.

Managers can view real-time disease data, generate statistical reports, and adjust prevention and control strategies based on the detection results through the backend. In addition, the backend supports flight path planning and management, intelligent pesticide application management, as well as data storage and query functions. It enables long-term tracking of disease occurrence trends and provides data support for the health management of tea plantations.

3.There is an error in the spelling of the caption for figure 4.

Author Response：

The English title of Figure 4 has been changed to "UAV flight attitude during aerial photography".

4.You say that the system was self-developed. Was the whole drone system + detection system was developed by yourself the development took place only at the level of the detection system which was then integrated within the off the shelf drone. This question was raised in my previous review as well. Please state clearly.

Author Response：

Thank you for the reviewer's correction! To clarify the scope of "self-developed", the "self-developed system" referred to in this paper mainly refers to the tea leaf disease detection system, including modules for image acquisition, model inference and deployment, and data processing and analysis. The UAV platform itself is an off-the-shelf agricultural UAV (DJI T25P). Based on this UAV, this paper conducts the integration and adaptation of the detection system, including the configuration of the Jetson TX2 edge computing module, OAK-D camera, GPS positioning interface, and data communication. The front and rear obstacle avoidance sensors, downward optical flow sensor, phased array radar, and flight control system are all original configurations of the UAV, which are used to ensure flight stability and safety. Therefore, the innovation and independent development work of this paper are mainly focused on the detection system level; the UAV platform itself is not self-developed but used as the foundation for integration. We have re-described this part to avoid misunderstandings among readers.

The revised text is as follows:

This paper proposes a tea leaf disease detection system developed based on the integration of an existing agricultural UAV platform, whose hardware composition is shown in Figure 2. The system takes the DJI T25P agricultural UAV as the carrier and integrates a high-performance Jetson TX2 edge computing module, an OAK-D camera, and a GPS module. Among them, the detection system part is self-developed, including modules for image acquisition, disease recognition model inference, data processing, and result uploading; the UAV platform (such as the flight control system, power system, and obstacle avoidance radar) is off-the-shelf equipment.

The OAK-D camera is the core image acquisition device of this system, equipped with a binocular depth camera and an RGB main camera, which can simultaneously output high-definition images and depth map information. This camera supports six-degree-of-freedom spatial perception capability and exhibits good image stability and robustness in the complex environment of tea gardens. Currently, the system adopts a single-camera design to balance the load and performance, but the system architecture supports horizontal expansion to a multi-camera array to meet the operational needs of large areas or multi-angle scenarios.

The Jetson TX2 is the core computing module, featuring high performance and low power consumption, and can complete tasks such as image preprocessing, model inference, and result uploading on the edge side. The GPS module is used for the precise positioning of aerial photography routes. The front and rear obstacle avoidance sensors, downward optical flow sensor, and phased array radar jointly ensure the stability and safety of the UAV in complex environments.

5.There is a mention of images collection but the phrase suggests images will be collected in the future. If that’s the case why the authors have decided to choose 3037 images. If they have already been collected please correct the grammar and also provide the reason for collecting 3037 images. This question was raised in the previous review.

Author Response：

Thank you for the reviewer pointing out the issue of semantic ambiguity! We have completed the image acquisition work, corrected the tenses in the paper, and supplemented the description of the number of collected data. A total of 3,037 tea leaf images were collected in this study, and the sample size was determined based on the following considerations:

In the experiment, the number of images collected was comprehensively calculated based on factors such as the tea plantation area (approximately 50,000 m²), UAV flight route spacing (10 m), flight altitude (10–15 m), and camera field of view (approximately 120°). The purpose was to ensure sufficient spatial coverage and redundancy of images while controlling the rationality of the data volume. Therefore, approximately 2,800–3,300 images were needed. Finally, by flying with these parameters and ensuring full coverage, a total of 3,037 images were collected. This number not only meets the sample diversity required for model training but also balances the efficiency of subsequent data annotation and processing.

The revised text is as follows:

A total of 3,037 original tea leaf images were collected. This quantity was determined comprehensively based on the experimental area (approximately 50,000 m²), UAV flight path planning (10 m spacing, 10–15 m flight altitude), and camera field of view (approximately 120 degrees). Calculated based on the area covered by a single UAV flight and an image overlap rate greater than 90%, approximately 2,800 to 3,300 images were theoretically required to achieve full-coverage imaging of the entire tea plantation area and multi-angle redundant sampling. Eventually, 3,037 original images were collected through actual field acquisition.

The images have a uniform resolution of 4032 × 3024 pixels and are stored in JPG format. Through a strict quality screening process based on criteria such as image clarity (greater than 0.8), lighting uniformity, and target integrity, 2,432 high-quality disease images were ultimately retained, achieving a screening rate of 80.1%. The distribution of various diseases in the dataset is relatively balanced: 641 images (26.4%) correspond to tea mosquito bug infestation—the largest class—and 142 images (5.8%) correspond to black rot—the smallest class. This distribution helps ensure balance and supports the generalization ability of model training.

6.The headings hierarchy is a bit confusing and it is hard to differentiate between the main heading and the sub-heading. E.g. ‘Methods’ versus ‘Yolo11 Network Structure’. Please check and distinguish between them carefully.

Author Response：

Thank you very much for the reviewer’s careful suggestion! We have thoroughly standardized the heading hierarchy and formatting throughout the manuscript, using font sizes to distinguish different levels.

7.I did not seem to find an answer to this question I raised - The drone seems to be significantly large with 26kg weight. How this aligns with your claim of compact size drone. These two seem to be contradictory.

Author Response：

Thank you for the reviewer's further correction! The use of the term "compact size drone" in the initial draft was mainly based on the following two considerations:①Relative classification criteria: DJI's current mainstream agricultural UAVs include the T25P (26 kg), T70 (56 kg), and T100 (73 kg). When comparing payload capacity, body size, and operational convenience, the T25P is a lightweight and miniaturized model in this series, so it is regarded as "compact" among similar agricultural UAVs;②Official manufacturer definition: According to the technical description on DJI's official website, the T25P adopts a foldable structure and a small-sized body design, with a 20 kg spraying capacity and 25 kg sowing capacity. It supports single-person loading/unloading and flexible transition operations, and is positioned as a small, flexible, and efficient agricultural UAV platform.

Therefore, the term "compact size drone" in this paper is based on the relative size and structural characteristics of this model among similar products, rather than a definition based on absolute weight.

8.This has not been addressed from my previous review: Explain the role of every method you have used and align it with your research questions and also the function you are achieving in a summary table to show how these different methods come together to achieve an overall outcome. To make it clear, I would suggest authors to include an outline/flow diagram or a table including all the approaches, showing a stage + approach in the flow and the function/purpose against each one of them to provide a bigger picture to the readers.

Author Response：

Thank you for the reviewer's valuable comments! In accordance with your suggestions, we have added a new subsection to the "Materials and Methods" section of the paper, which systematically organizes the various methods used in this study and clearly explains the function, purpose of each method as well as its relationship with the research objectives. Meanwhile, we have added "Table 3: Main Methods Employed in This Study and Their Corresponding Functions". These revisions have improved the clarity of the paper's structure and logical coherence, making it easier for readers to understand the research framework of this paper.

The revised text is as follows:

Method Summary and Process Description

| Method name | Stage | Main function and role | Problems solved | Relationship with research objectives |
| --- | --- | --- | --- | --- |
| UAV low-altitude remote sensing image acquisition | Data acquisition stage | Achieve data collection covering whole tea plantation areas via high-resolution UAV aerial photography | Low efficiency and insufficient coverage of traditional manual collection | Provide comprehensive, high-quality image data foundation for model training |
| Data augmentation (brightness adjusting, rotation, cropping, mosaicing) | Data preprocessing stage | Expand sample diversity and improve model generalization ability | Limited data volume and susceptibility to overfitting | Enhance model robustness and stability |
| FC_C3K2 module (FasterNet + CGLU) | Backbone | Extract lightweight feature, and improve feature expression capability in complex backgrounds | Complex backgrounds and difficult feature separation | Enhance the model's ability to extract tea leaf disease features |
| HSFPN module | Neck | High-level screening and multi-scale feature fusion | Poor performance in small target detection | Improve detection accuracy and robustness for diseases at different scales |
| Efficient-Head structure | Head | Use GConv to reduce parameter count and computational complexity | Slow inference speed and overfitting | Improve real-time detection performance and edge deployment efficiency |
| Model evaluation and comparative experiment | Performance verification stage | Validate the improvements by comparing with mainstream models | Lack of systematic verification | Demonstrate the comprehensive advantages of the proposed model in accuracy, speed, and lightweight design |
| Edge deployment | Model deployment stage | Deploy on Jetson TX2 platform and calculate model inference efficiency and power consumption | Limited real-time detection performance | Verify the practical deployment feasibility of the model's lightweight design |

9.The following also relates to my previous comment. The network structure you ave presented in Figure 6 (now 7) for YOLO is already quite complicated for a general reader. You must provide a high-level overview with clear names and labels before diving into a detailed structure. If you have provided this already, indicate where it is in the paper?

Author Response：

Thank you for your valuable suggestions! We have added a high-level overview of the YOLO11 network structure at the beginning of the "YOLO11 Network Structure" section, which provides a concise description of the overall network—including its three main modules (Backbone, Neck, and Detection Head) and their core functions—to help general readers gain a clear understanding of the network structure before delving into details. Meanwhile, the internal structure and functions of each module are elaborated on in subsequent paragraphs. The high-level overview is clearly marked at the start of the "YOLO11 Network Structure" section and is accompanied by Figure 7, which illustrates the overall network architecture. The structures of modules such as SPPF, C2PSA, C3K2, C3, and Detect are labeled in the upper right corner of Figure 7, while the rest are basic deep learning modules.

The revised text is as follows:

**YOLO11 Network Structure**

YOLO11 is the latest version in the YOLO series, optimized from YOLOv8 to achieve better performance on the COCO dataset with fewer parameters. The network structure of YOLO11 is shown in Figure 7, which specifically comprises three core modules: backbone network, feature fusion layer, and detection head. The Backbone is responsible for extracting multi-scale features from input images, the Neck fuses features of different scales to enhance detection performance, and the Detection Head converts the processed feature maps into target bounding boxes and category predictions, enabling the final detection results.

The Backbone is the fundamental component of YOLO11, and its main structure includes the Conv (Convolutional Layer) module, C3K2 module, SPPF (Spatial Pyramid Pooling Fast) module, and C2PSA (Cross-stage Partial Spatial Attention) module, ensuring the model can efficiently extract local and global features. Firstly, the Conv (Convolutional Layer) module is the initial part of the backbone network, responsible for basic low-level feature extraction of input images and providing a foundation for subsequent high-level feature extraction. Next, the C3K2 module integrates multiple convolutional layers and bypass connections, and adopts flexible convolutional configurations and bottleneck designs to improve feature extraction capability. By introducing the Cross Stage Partial (CSP) architecture, the C3K2 optimizes gradient flow, enhances local feature extraction, improves training stability, and strengthens the robustness of YOLO11 in complex backgrounds. Finally, the SPPF (Spatial Pyramid Pooling Fast) module enhances the model’s ability to capture global contextual information through multi-scale pooling operations, while the C2PSA (Cross-stage Partial Spatial Attention) module improves the model’s detection ability in complex scenes by effectively filtering irrelevant information and emphasizing key features—exhibiting significant advantages especially in multi-target detection and occlusion problems.

The Neck module connects the Backbone and Detection Head, responsible for the fusion of multi-scale features and particularly suitable for detecting objects of different sizes. Its core task is to enhance object detection performance and ensure the model maintains efficient performance across multiple scales. Firstly, the Upsample (Up-sampling) module helps the model better detect smaller objects by increasing the resolution of low-resolution feature maps. Secondly, the Concat (Feature Concatenation) module fuses feature maps with different resolutions, enabling the model to utilize multi-scale information to improve detection accuracy. Finally, the C3K2 module in the Neck enhances feature representation capability and improves detection accuracy by performing repeated convolution operations and shortcut connections for cross-scale feature fusion. The introduction of the C3K2 module allows the model to achieve stronger expression ability across different scales, thereby improving overall detection performance.

The Detection Head is the final stage of YOLO11, responsible for converting processed feature maps into accurate object detection results. With its multi-scale capability, it ensures the model exhibits excellent performance in object detection tasks involving targets of various sizes. Firstly, the classification detection head adopts the Depthwise Separable Convolution (DWConv) structure—a lightweight convolution method that improves computational efficiency by simplifying convolution operations. Through the combination of depthwise convolution and pointwise convolution, DWConv reduces computational cost while maintaining high detection accuracy. Next, the bounding box detection path captures precise spatial information through standard convolution, ensuring accurate prediction of target boundaries. Finally, the classification detection path utilizes DWConv for classification tasks, reducing computational complexity and making it particularly suitable for resource-constrained environments such as edge devices or embedded systems. While ensuring accuracy, DWConv significantly reduces computational cost, enabling YOLO11 to efficiently handle multi-target scenes.

10.Authors mentioned sections 2.2 and 3.2 but where are they in the paper? None of the sections in this paper are numbered. How a reader will know what part of the paper is 2.2 or 3.2, etc.?

Author Response：

Thank you for pointing this out. Sections 2.2 and 3.2 were the paragraph markers before I formatted the paper according to the PLOS ONE format. I have removed them in the new format and made the corresponding changes in the paper.

11.This comment has not been addressed: Results in Table 4 and other tables must highlight the top three models. Label your model as (Our) in the table along with the name.

Author Response：

Thank you for your valuable suggestions! We have highlighted the first three models in bold in all tables and labeled them with "(Our)" to emphasize them. All tables have also been uploaded for easy reference.

12.Figure 17, the x/y axis have not been properly labelled and does not provide explanation of what this figure is about, when looking at the figure itself without referring to the text.

Author Response：

Thank you for your valuable suggestions! We have redrawn the X/Y axes in Figure 17.The specific modifications are shown below:

**Fig 17. Dataset visualization**

13.Table 5, add (our) against your model(s).

Author Response：

Thank you for your suggestion! We have marked "(Ours)" in all tables to highlight our model.

14.The authors may consider finding a better way to represent the images from all the model results (Fig. 22-32) since they are the figures presented one after the other in a row which is a bit odd for the paper structure.

Author Response：

Thank you for your valuable suggestion! We have arranged Figures 22-32 horizontally to display all model results more clearly. The revised example is shown in Figure 22.

**Fig 22. Heatmap Comparison Figure**

15.Indicate where this is in the paper? Provide F1 score results for the combined precision and Recall.

Author Response：

Reply: Thank you for your valuable suggestion! Firstly, we have provided the calculation formula of the F1 score and its meaning in the "Evaluation Metrics" section. Secondly, we have added the F1 score values to both Table 5 and Table 6, and conducted a comparative analysis.
